# Tankyrases as modulators of pro-tumoral functions: molecular insights and therapeutic opportunities

**DOI:** 10.1186/s13046-021-01950-6

**Published:** 2021-04-28

**Authors:** Esteban Zamudio-Martinez, Ana Belén Herrera-Campos, Alberto Muñoz, José Manuel Rodríguez-Vargas, F. Javier Oliver

**Affiliations:** 1 Instituto de Parasitología y Biomedicina López Neyra, CSIC, CIBERONC, 18016 Granada, Spain; 2 Centro de Investigación Biomédica en Red de Cáncer, CIBERONC, 28029 Madrid, Spain; 3 Instituto de Investigaciones Biomédicas “Alberto Sols”, CSIC, Universidad Autónoma de Madrid, 28029 Madrid, Spain

**Keywords:** TNKS1/2, Tankyrase binding motif, Proteasomal degradation, Scaffolding function, Cancer, Inhibitors

## Abstract

Tankyrase 1 (TNKS1) and tankyrase 2 (TNKS2) are two homologous proteins that are gaining increasing importance due to their implication in multiple pathways and diseases such as cancer. TNKS1/2 interact with a large variety of substrates through the ankyrin (ANK) domain, which recognizes a sequence present in all the substrates of tankyrase, called Tankyrase Binding Motif (TBM). One of the main functions of tankyrases is the regulation of protein stability through the process of PARylation-dependent ubiquitination (PARdU). Nonetheless, there are other functions less studied that are also essential in order to understand the role of tankyrases in many pathways. In this review, we concentrate in different tankyrase substrates and we analyze in depth the biological consequences derived of their interaction with TNKS1/2. We also examine the concept of both canonical and non-canonical TBMs and finally, we focus on the information about the role of TNKS1/2 in different tumor context, along with the benefits and limitations of the current TNKS inhibitors targeting the catalytic PARP domain and the novel strategies to develop inhibitors against the ankyrin domain. Available data indicates the need for further deepening in the knowledge of tankyrases to elucidate and improve the current view of the role of these PARP family members and get inhibitors with a better therapeutic and safety profile.

## Background

**Fig. 1:**
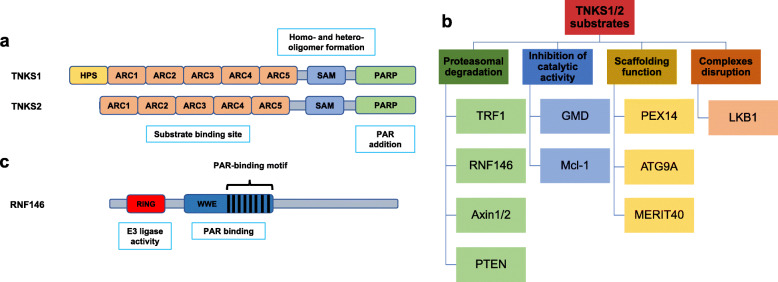
TNKS1/2 and RNF146 structure and functional classification of tankyrase substrates. Both tankyrases share a similar structure that consist in the C-terminal catalytic PARP domain that catalyze the synthesis and addition of linear PAR chains onto their substrates, the SAM domain responsible for the formation of multimeric structures with other tankyrases and the ankyrin domain divided in five ankyrin domain clusters (ARC1–5) which mediate the recognition and binding to the substrates (except for ARC3). In the case of TNKS1 there is also an HPS region with unknown function (**a**). The substrates of tankyrase can be classified into four categories according to the biological response that trigger when they interact with TNKS1/2: proteasomal degradation, cataytic inhibition, scafffolding function and complexes disruption (**b**). RNF146 presents two characterized domains in the N-region: the RING domain responsible for the E3-ligase activity and the WWE domain, which recognizes the iso-ADP-ribose moiety present in the PARylated substrates via its PAR-binding motif (**c**)

Tankyrase 1 (TNKS1) and tankyrase 2 (TNKS2) are two homologous proteins that belong to the Poly (ADP-ribose) polymerases (PARPs) family (also renamed ARTDs), a group of 17 members that catalyze the addition of ADP-ribose molecules into a large and variety amount of proteins, causing a reversible posttranslational modification (PTM) commonly named ADP-ribosylation [1, 2]. TNKS1 and TNKS2 share an 81% nucleotide homology and 85% amino acid identity [3]. *TNKS* is located on chromosome 8, contains 1327 amino acids and its primary structure can be classified into four different domains: The C-terminal catalytic PARP domain that mediates the PAR addition to its substrates, an sterile alpha module (SAM) responsible for the homo- and hetero-oligomer formation, the ankyrin (ANK) domain divided in 5 clusters (ARC 1–5) which serve as the substrate binding site, and the His, Pro and Ser (HPS) rich domain with unknown function at the N-terminus [4, 5]. The sequence of HPS domain presents a low complexity, suggesting that it is intrinsically disordered [6]. *TNKS2* is situated on chromosome 10, contains 1166 amino acids and presents a similar organization to *TNKS*. Both catalytic domains are highly conserved, with 94% homology; the SAM domain has 74% similarity and the ANK domain shares 83% identity. However, TNKS2 present a different N-terminal region, lacking the HPS domain [5] (Fig. 1a). To date, the FDA (Food and Drug Administration) has approved the use of four PARP inhibitors as a treatment for different tumors: olaparib, rucaparib, niraparib and talazoparib, which have shown a high effectiveness and reduced adverse effects in cases of breast, ovarian and prostate tumors with *BRCA1/2* mutations [7, 8]. New groups have focused their projects on the biochemical and functional study of other less characterized members of PARP family, whose clinical potential is in full expansion. Of all of them, the Tankyrase group has a high therapeutic potential in various human diseases. The present review discusses Tankyrases function and the current state of development of chemical inhibitors and their potential as therapeutic targets in cancer.

### Tankyrases: the diversity of biological functions and substrates

TNKS1 has been the best studied of the two isoforms due to its greater abundance. Nevertheless, since their discover, tankyrases have been found to be involved in several functions [9] such as telomere elongation [10], Wnt/β-catenin signaling [11], mitosis [12], glucose metabolism [13], pexophagy [14] and DNA repair [15]. Tankyrases interact with all their substrate proteins through the ANK domain, which contains 24 ankyrin repeats that are organized into 5 subdomains, commonly called ankyrin repeat clusters (ARC1–5) [16–18]. It has been described that each ARC works as the basic unit for the recognition of peptides present in tankyrase substrates [19, 20]. ARCs properties have been studied using peptides and it has been shown that ARC1, ARC2, ARC4 and ARC5 are able to interact with tankyrase partners with a similar binding mode. However, ARC3 maintains a poor conserved binding surface and therefore, it does not bind tankyrase substrates [6, 21]. In fact, more studies are needed to elucidate the ARC3 function, since it might help the rest of ARCs to the full-length protein binding. TNKS1 and TNKS2 seem to have most binding partners in common and a similar localization, including Golgi complex [22], cytoplasm [23], peroxisomes [14], telomeres [24], centrosomes and nuclear pores [25]. Therefore, TNKS1 and TNKS2 may share most of their functions and one tankyrase could substitute for one another, although it has also been shown that both tankyrases are required to maintain some specific functions [23]. An amino acid sequence present in most tankyrase substrates has been identified. This sequence is called Tankyrase Binding Motif or TBM and Guettler and colleagues defined the consensus TBM as a sequence of eight residues RXXPDGXX. However, another kind of TBM different to the consensus or canonical TBM has been described. These non-canonical TBMs contain additional amino acids between the key arginine and glycine residues, but they still behave and bind to the ANK domain of TNKS1/2 in a similar way to the canonical TBM. This information adds new possibilities to the concept of TBM and increases the number of possible tankyrase substrates. Some of them like Axin1 or RNF146 present both kinds of TBMs (canonical and non-canonical). Nevertheless, other proteins like NELFE and IF4A1 only contain non-canonical TBM to interact with TNKS1/2 and we were not considering them as a target [6, 26]. The TBM also seems to bethe same recognition signal by the four active ARCs [17, 27]. As a member of PARP family, tankyrases can mediate the addition of linear poly (ADP-ribose) (PAR) chains onto their substrates. The linear chains of ADP-ribose are negatively charged and this effect can alter the properties of tankyrase target acceptors [28]. In comparison to the rest of ARTDs, tankyrases do not contain the α-helical regulatory domain that controls the catalytic activity. However, several studies have elucidated the role of tankyrase domains in order to regulate its catalytic activity. The SAM domain allows the formation of large molecular TNKS1/2 complexes, which enhance the PARP activity of tankyrase [29, 30]. TNKS1 can dissociate these complexes through autoPARylation, thus, regulating its own catalytic activity. The ANK domain also helps selecting TNKS1/2 binding partners to be PARylated. This fact is supported by the idea that each active ARC has micromolar affinity for tankyrase substrates and the spatial position of each ARCs is essential for the binding mode of tankyrase with its targets. A model based on crystallography and small-angle-X-ray scattering (SAXS) showed that ARC1, ARC2 and ARC3 present a rigid conformation, with a broken helix between ARC1 and ARC2 and a continuous helix between ARC2 and ARC3. Conversely, ARC4 and ARC5 present a more flexible conformation in comparison to the ARC1–3 module. ARC2 and ARC4 present high affinity for binding target peptides, while ARC1 and ARC5 show low affinity. All this information point out the ANK domain of tankyrase as a multivalent binding system with an special structure that explains how all ARCs collaborate in order to interact with the target partners and also the position of tankyrases to PARylate their substrates. The information about the specific interaction between Axin1 and TNKS1 gave the idea that the binding mode of Axin1 promotes its PARylation because its optimal orientation brings the catalytic domain closer to the target protein. The presence of one or more TBM and their spatial positioning could modulate the interaction with TNKS1 and enhance its catalytic activity [6, 31] The recognition of the TBM by TNKS1/2 triggers diverse biological responses including proteasomal degradation, tankyrase catalytic inactivation, scaffolding function or complexes disruption [17] (Fig. 1b and Table 1).

**Table 1:** Tankyrase binding motifs of tankyrase substrates

Substrate	PARylated	Biological response	TBM	References
**TRF1**	Yes	Proteasomal degradation	RGCADG	[27, 32]
**3BP2**	Yes	Proteasomal degradation	RSPPDG	[17]
**RNF146**	Yes	Proteasomal degradation	TBM1: RESSADG TBM3: RSHRGEG TBM4: RSVAGG TBM5: RSRRPDG	[26, 33]
**Axin1**	Yes	Proteasomal degradation	TBM1: RPPVPG TBM2: RRSDLDLGYEPEG	[6]
**PTEN**	Yes	Proteasomal degradation	RYQEDG	[34]
**Mcl-1**	No	Inhibition of TNKS1/2 catalytic activity	RPPPIG	[35]
**GMD**	No	Inhibition of TNKS1/2 catalytic activity	RGSGDG	[32, 36]
**PEX14**	No	Scaffolding function	TBM1: RMEVQG TBM2: RRGGDG	[14]
**ATG9**	No	Scaffolding function	RLPGLG	[14]
**MERIT40**	Yes	Scaffolding function	TBM1: RSNPEG TBM2: RSEGEG	[37, 38]
**LKB1**	Yes	Complexes disruption	TBM1: RAKLIG TBM2: RRIPNG	[39]

#### Proteasomal degradation

**Fig. 2:**
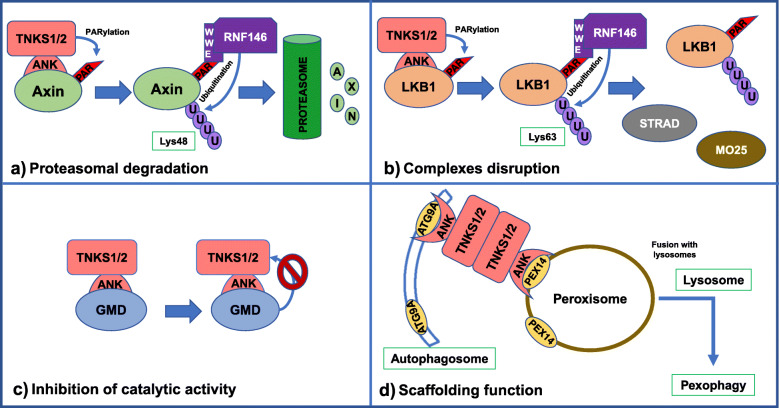
Biological responses triggered after the recognition of TNKS1/2 substrates. Tankyrases recognize their binding partners via the ANK domain. The most studied function of TNKS1/2 is the regulation of protein stability via proteasomal degradation. Tankyrases PARylate many substrates like Axin1/2, PTEN or 3BP2 and then, the E3 ubiquitin ligase RNF146 recognizes the PARylated proteins and adds a poly-ubiquitin chain linked by its Lys48 residues, which trigger their proteasomal degratation (**a**). More recently, LKB1 was shown to interact with TNKS1/2 and RNF146, but in this case, tankyrase is able to PARylate LKB1 and RNF146 adds a poly-ubiquitin chain linked by Lys63 in orde to disrupt the complex formed by LKB1, STRAD and MO25 (**b**). The interaction between tankyrase and the proteins GMD and Mcl-1 causes the inhibition of the catalytic activity of TNKS1/2 (**c**). Tankyrases also can work as scaffolding proteins via their ANK domain, promoting the interaction between different proteins like PEX14 and ATG9 (without PARylation) or MERIT40 (low levels of PAR) (**d**)

One of the best characterized functions of tankyrases is the regulation of protein stability via proteasomal degradation. PARylation by tankyrase is tightly linked to ubiquitination by an E3 ubiquitin ligase called RNF146 (RING finger protein 146) or Iduna. Tankyrases recognize their binding partners and PARylate them through their ANK domain and PARP domain, respectively. Then, RNF146 recognizes PARylated proteins by its WWE domain, which specifically recognizes the iso-ADP-ribose moiety present only in PAR chains (Fig. 1c). RNF146 catalyzes the formation of a poly-ubiquitin chain linked by its Lys48 residues, in order to trigger the degradation of the substrate by the 26S proteasome. This process is commonly called PAR-dependent ubiquitination or PARdU and was discovered through the implication of RNF146 and TNKS1/2 in the degradation of Axin1 [40–42]. Several groups have shown that a chain of at least four Lys48-linked ubiquitins is necessary to achieve proteasomal degradation [21]. Up to now, it has been described that the stability of many proteins are regulated via PARdU (Table 1), including Axin1/2 [40], PTEN [34], 3BP2 [17], TRF1 [24] or even TNKS1/2 and RNF146, since both are substrates of one another [41, 43] (Fig. 2a).

##### TRF1

The telomeric repeat-binding factor 1 or TRF1 is a member of the six-subunit shelterin complex responsible for the protection and replication of telomeres. This factor contains a TBM indispensable for the recognition by tankyrase [27, 32] (Table 1). A crystal structure of TRF1-TNKS1(ARC2–3) complex revealed additional interactions between the ANK domain of TNKS1 and flanking residues of the TBM present in TRF1, indicating not only the importance of TBM, but surrounding residues to the TNKS1-TRF1 interaction [44]. The role of TRF1 and tankyrase has been related to different functions. Firstly, tankyrase was discovered as a positive regulator of telomerase due to its implication in the releasing from telomeres and degradation of TRF1 [5]. TRF1 is considered a telomerase inhibitor. It has been described that the loss of TRF1 by TNKS1/2 leads to the telomerase upregulation and thus, telomeres elongation [28]. Tankyrase 1 has also been involved in resolving sister telomeres at mitosis because TRF1 and TIN2, another member of shelterin complex, are associated to SA1, one subunit of the cohesin complex. Cohesins are the proteins that maintain sister chromatids together from their replication in S phase until mitosis, when they are segregated to daughter cells. TNKS1 is responsible for the TRF1-TIN2-SA1 dissociation via TRF1 PARylation, allowing sister telomeres separation [12, 45]. More recently, TNKS1 has been implicated in the DNA Damage Response (DDR) mechanism at telomeres. Specifically, when an oxidative DNA damage causes a Single Strand Break (SSB) in telomeres, TNKS1 is recruited to the area via TRF1 interaction. Then, TNKS1 PARylates TRF1 and the linear poly (ADP-ribose) chain acts as a primary signal to recruit proteins implicated in Single Strand Break Repair (SSBR) like XRCC1 and Pol-β. Despite PARP1 has been considered the key master of DDR mechanism, the inhibition or silencing of PARP1 does not alter the recruitment of XRCC1 to the damaged telomeres [15]. This research suggests a new mechanism for TNKS1 in the initial phase of SSBR at telomeric sites, which contributes to genome stability, cell survival and is independent of cell cycle and telomerase.

##### RNF146

RING finger protein 146 (RNF146) or Iduna is a RING E3 ubiquitin ligase responsible for the protein ubiquitination. This posttranslational modification is a tight regulated process and is implicated in a large variety of cellular functions carried out by a complex of proteins. It is necessary an E1 ubiquitin activating enzyme, an E2 ubiquitin conjugating enzyme and an E3 ubiquitin ligase, which confers the substrate specificity [46]. RNF146 structure can be divided in two parts: the N-terminal region and the C-terminal region. The N-terminal region contains two characterized domains, the WWE domain and the RING domain, which control the PAR binding function and the E3 ligase activity, respectively (Fig. 1c). The C-terminal domain seems to be intrinsically disordered, which consist in a lack of a fixed three dimensional structure [33, 47]. The WWE domain is also present in some PARP family members, although is less studied. For example, the WWE domain of PARP11 has been described to interact with the ADPr subunit, while the WWE domain of RNF146 recognizes the iso-ADP-ribose moiety, the shortest internal unit present only in PAR chains [42, 48, 49]. RNF146 function is closely related to the PARylation by Tankyrases through the process of PARdU [40, 41]. Once the target protein is PARylated, RNF146 recognizes and binds to iso-ADPr unit via its WWE domain. It has been shown that the iso-ADPr moiety also interacts with the RING domain. The ligand binding leads to a conformational change that activates RING domain and this modification results in the allosteric switch that increases the E3 ligase activity of RNF146. This mechanism explains how both WWE and RING regions collaborate to regulate the catalytic activity of RNF146 [33]. Moreover, RNF146 is another substrate of tankyrase. Five possible TBM were observed in the disordered C-terminal region of RNF146 (TBM 1–5), although the TBM4 is the only canonical TBM, while the rest of candidates (TBM 1–3 and 5) are one or two residues longer than the canonical TBM (Table 1). According to a recent study, TBM 1 and 4 seem to be the most important for the TNKS1-RNF146 interaction, but the largest effect on TNKS1 binding was found when TBM1 was mutated and not TBM4, which is a consensus TBM. TBM 3 and 5 also present additional contributions to the interaction between tankyrase and RNF146. Nevertheless, TBM2 does not appear to contribute to the interaction with TNKS. This study revealed a new TBM model that presents one extra residue in the middle of the canonical TBM sequence. The RNF146 (TBM1) – TNKS1 (ARC2–3) interaction model showed that extended TBM binds to ARC2 with an affinity comparable to consensus TBMs. Furthermore, the RNF146-TNKS1 (ARC1–5) interaction model supports the idea of RNF146 using multiple motifs to binding multiple ARCs of tankyrase simultaneously [26].

##### Axin1/2

Axis inhibition 1 and 2 (Axin1/2) are considered negative regulators of the canonical Wnt pathway [50, 51]. β-catenin is the key master of Wnt signaling and is tightly regulated by the β-catenin destruction complex, which is composed by APC, CK1α, GSK3β and Axin, being the last one the rate-limiting factor. This complex mediates the phosphorylation of β-catenin, which then is recognized and ubiquitinated by the ubiquitin ligase SCF^β-TrCP^ in order to target it for proteasomal degradation [52]. Axin was the first well-defined target of PARdU, since TNKS1/2 and RNF146 mediate its degradation via proteasome [11, 40, 41, 47]. For this reason, TNKS1/2 are considered positive regulators of the canonical Wnt pathway. Two TBMs have been described into the Axin1/2 amino acid sequence. One of them is a canonical TBM, while the other one is a non-canonical TBM, with four additional residues between the key Arg and Gly residues (Table 1). These TBM are conserved in the Axin family and between species. Several crystal structures analysis combined with mutational assays between TNKS1 and Axin1 have demonstrated that both TBM are necessary to mediate a bivalent interaction with the ANK domain of tankyrase and regulate Axin turnover [6, 50].

##### PTEN

Phosphatidylinositol (3,4,5)-trisphosphate phosphatase or PTEN is an enzyme that acts as a negative regulator of the PI3K-Akt signaling pathway [53]. PTEN mediates the dephosphorylation of phosphoinositides to antagonize the kinase PI3K [54]. This pathway controls a variety of processes related to cell cycle and cellular proliferation, thus PTEN is considered a tumor suppressor [55]. PTEN activity is regulated by several posttranslational modifications such as acetylation, ubiquitination, SUMOylation or phosphorylation [56–58]. These modifications are able to control the stability of the enzyme, since some studies revealed the implication of phosphorylation in the regulation of the ubiquitin-mediated degradation [53, 59]. Recently a new modification of PTEN, TNKS1/2-dependent PARylation, has been discovered. PTEN contains a TBM at the N-terminal region, close to the phosphatase domain (Table 1). It has also been shown the implication of RNF146 in the recognition of PARylated PTEN and its ubiquitination and degradation via proteasome. An in vitro ribosylation assay of PTEN by both TNKS1/2 detected three different PAR acceptor sites by tankyrases (Glu40, Glu150 and Asp326). Furthermore, in vivo ubiquitination assays with mutations in different lysine residues of PTEN showed that the major sites for PTEN ubiquitination mediated by RNF146 are Lys342, Lys344 and Lys349 [34].

#### Other biological responses triggered after the interaction with tankyrases

Despite our knowledge about tankyrases is increasing, some aspects remain unclear. One of them is how regulatory domains control TNKS1/2 catalytic activity. The ANK domain of TNKS1/2 is responsible for the interactions with a large variety of functional and structural binding partners [6, 32]. Most TNKS1/2 substrates have been shown to be PARylated. Moreover, the most characterized biological response triggered after PARylation is the recognition by the E3 ubiquitin ligase RNF146, which triggers the Lys48-linked ubiquitination and the proteasomal degradation of the target [43]. However, several evidences suggest this is not the only biological response. A recent study revealed LKB1 as a new tankyrase binding partner that after being PARylated, it was recognized by RNF146, but it was not degraded [39]. Other proteins like Mcl-1 [35], PEX14, ATG9A [14], MERIT40 [37] and the enzyme GDP-Mannose-4,6-Dehydratase [36] are also able to engage TNKS1/2 without being modified, or even can inhibit the catalytic activity of tankyrase, indicating that different parameters determine TNKS1/2 interaction and/or PARylation (Fig. 1b).

#### Complexes disruption

Liver kinase B1 or LKB1 is a serine/threonine kinase implicated in cellular energy homeostasis and is also the major upstream activator of AMPK [60]. LKB1 regulates the activation of AMPK through its phosphorylation at Thr172 [61]. Activated p-AMPK is able to phosphorylate downstream targets implicated in glycolysis and protein and fatty acid synthesis [62–65], causing a decrease in cell proliferation under normal conditions, although increases cell survival during energy stress conditions [66]. LKB1 presents a weak catalytic activity in vitro and in vivo*,* and its activation is promoted by the formation of a complex with the proteins STRAD and MO25 [67]. The binding of STRAD to the kinase domain of LKB1 promotes its kinase activity [68] and MO25 increases the effect of STRAD on LKB1 [69]. Phosphorylation of LKB1 at several residues has been associated to different functions. For instance, the phosphorylation at Ser428 is related to the AMPK activation [70]. Recently, LKB1 was shown to be a new tankyrase partner since the inhibition or the double loss of TNKS1/2 but not PARP1/2 inhibition enhances the phosphorylation of LKB1 at Ser428, the phosphorylation of AMPK and the expression of their target genes. Two possible tankyrase-binding motifs were found in the amino acid sequence of LKB1 (Table 1). Both TBMs are highly conserved and are indispensable for the interaction with TNKS1/2. In vitro ribosylation assays along with mass spectrometry and point mutations assays determined that Glu130 and Glu138 are the residues critical for LKB1 PARylation. RNF146 also works together with TNKS1/2 to regulate AMPK activation recognizing PARylated LKB1. However, the loss of RNF146 enhances the phosphorylation of LKB1 at Ser428, the activation of AMPK, but does not alter LKB1 protein levels. An in vivo ubiquitination assay proved that in the case of LKB1, RNF146 does not catalyze the Lys48-linked ubiquitination, which usually triggers proteasomal degradation. Instead, RNF146 regulates AMPK activation via non-degradative mechanism that involves Lys63-linked ubiquitination of LKB1. Crystallography analysis of LKB1 revealed that Glu130 and Glu138 residues are situated near the binding site of LKB1 with STRAD and close to the ATP binding groove of LKB1, respectively. All this information indicates that PARylation of theses residues and subsequent Lys63-linked ubiquitination prevent the formation of the LKB1/STRAD/MO25 complex and suppress the kinase activity of LKB1 (Fig. 2b). Therefore, TNKS1/2 and RNF146 modulate the catalytic activity of LKB1 and AMPK activation through the regulation of both complex formation and phosphorylation of LKB1, implicating a new mechanism of PARdU [39].

#### Inhibition of TNKS1/2 catalytic function

GDP-Mannose-4, 6-Dehydratase or GMD is an enzyme necessary for de novo synthesis of fucose, a monosaccharide present in several glycoproteins and glycolipids and also the major donor in all fucosylation reactions [71, 72]. GDP-fucose synthesis is carried out by GMD in the cytoplasm and transported to the endoplasmic reticulum or Golgi apparatus in order to be transferred to acceptor targets related to several biological functions such as leukocyte trafficking, neuron morphology and development [73]. GMD was identified as a tankyrase 1 partner, providing the existence of the association between both proteins during interphase. The analysis of GMD sequence revealed a highly conserved TBM present at the N-terminus (Table 1), which has shown to be essential for interaction with TNKS. Nevertheless, in vitro PARP assays and in vivo analyses probed that GMD is unable to be PARylated by tankyrase. In fact, the binding of GMD to TNKS suppresses its catalytic PARP activity. GMD depletion increases TNKS degradation via proteasome, indicating the influence of GMD in tankyrase autoPARylation and tankyrase-mediate PARylation of its targets. This inhibition is also specific for tankyrase and not for other PARPs like PARP1, since it requires the presence of the TBM in GMD [32, 36]. This enzyme represents a type of substrate that is not PARylated after binding to TNKS and besides inhibits its PARP catalytic activity (Fig. 2c). Indeed, this is not the only one described because a previous study detected another protein with a similar behavior. Mcl-1 (myeloid cell leukemia-1) is a member of Bcl-2 family implicated in apoptosis regulation [74]. The full-length protein (Mcl-1 L) has been identified as an antiapoptotic factor, while its short splicing variant (Mcl-1S) presents proapoptotic functions [75]. Both isoforms are substrates of TNKS and contain an identified TBM (Table 1), although they are not PARylation acceptors. Mcl-1 is able to inhibit the catalytic activity of TNKS, but to a lesser extent than GMD. Unlike GMD, it is not clear if the ability of Mcl-1 inhibition depends on its TBM [35]. Whether both partners share the same mechanism of inhibition or is mediated by different factors has to be determined.

#### Scaffolding function

The role of tankyrases in the cellular homeostasis is not only defined by the catalytic ability to PARylate a great number of proteins. TNKS1/2 can form multimeric structures with other TNKS1/2 via the SAM domain, acting as scaffolding proteins which promote the interaction between different substrates of tankyrases through the binding of their multiple targets to the ankyrin domain. The first evidence of this new role was the observation that tankyrases also participates in Wnt/β-catenin pathway independently of the PARP activity [31, 76, 77]. Furthermore, a proteomic assay of protein complexes associated to TNKS1/2 in both normal and under catalytic inhibition, revealed the interaction with two proteins implicated in pexophagy, PEX14 (Peroxin-14) and ATG9A (Autophagy-related protein 9A) [78–80]. PEX14 is a peroxisomal membrane protein that forms with another protein called PEX5 a heterodimer implicated in cytosolic material docking and delivery into the lumen of peroxisomes [81, 82]. ATG9A is the only transmembrane protein described among the ATG family and it is required for the formation of autophagosomal structure [83, 84]. The analysis of the amino acid sequence of PEX14 revealed four potential TBMs, but only two of them are responsible for binding PEX14 to both tankyrases (Table 1), suggesting that these TBMs cooperate to mediate the interaction with TNKS1/2. Unlike PEX14, only one of the three possible TBMs discovered in the sequence of ATG9A is indispensable for its association with TNKS1/2 (Table 1). In addition, an essay with a PAR-inactive mutant of tankyrase showed that its enzyme activity is not necessary for the interaction with both pexophagy-related proteins. According to all the results obtained, TNKS1/2 work as scaffolding proteins that mediate the interaction between the peroxisomal protein PEX14 and the autophagy-related protein ATG9A (Fig. 2d). The formation of ATG9A-TNKS1/2-PEX14 complex induces pexophagy in a tankyrase dependent manner, but not depending on its PARP activity, since TNKS1/2 do not PARylate either of two proteins. The E3 ubiquitin ligase RF146, which is tightly linked to TNKS1/2 through the PARdU mechanism, is neither involved in this process of pexophagy. Therefore, the new mechanism described is a non-canonical and a ubiquitination-independent process for pexophagy [14]. More recently, MERIT40 (Mediator of Rap80 Interactions and Targeting 40 kD) has also been reported to be directly associated with tankyrase. Two possible TBM were found in the amino acid sequence (Table 1), although it was shown that TBM1 mainly mediates the interaction with TNKS1/2. The PAR level detected in MERIT40 was weaker compared to those of other tankyrase substrates and the overexpression of tankyrase did not cause the downregulation of MERIT40. In fact, it has been observed that the complexes formed by MERIT40 and TNKS1/2 work as scaffolds that control the DNA damage response (DDR) machinery and the spindle structure and function [37, 38].

### TNKS1/2 inhibitors in cancer

**Fig. 3:**
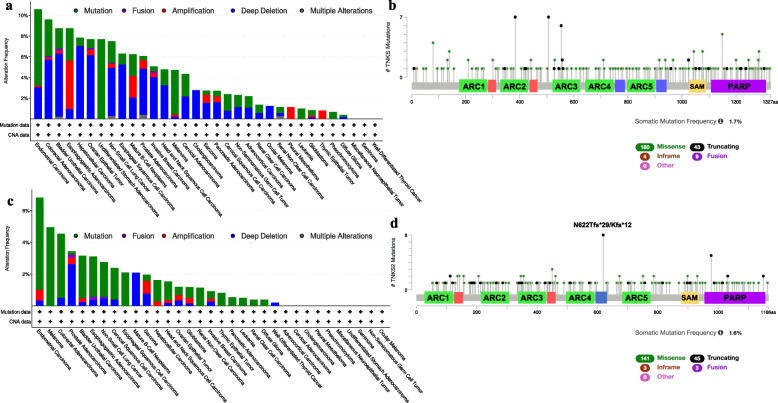
TNKS1/2 alterations in cancer. The bar graphics show the percentage of alterations for *TNKS* and *TNKS2* genes in multiple kinds of tumors (**a** and **c**) while the lollipop graphs show the mutation profile allong the sequence of both tankyrases genes (**b** and **d**) according to the TCGA PanCancer Atlas Studies from the cBioportal website

Tankyrases have such a broad range of substrates, that their alteration (mutation, up- or downregulation) is related to multiple diseases, including obesity [85], diabetes, fibrosis [86], Epstein Barr and Herpes simplex viral infections [87, 88], Cherubism [17], sclerosis [89] and cancer. Through the analysis of the public cancer portal cBioportal we have obtained a profile of the main cancer types affected by alterations in *TNKS/2* genes [90, 91]. For both genes, endometrial is the one that presented the highest rate of *TNKS* and *TNKS2* mutations (Fig. 3a and c), followed by colorectal cancer, bladder and esophagogastric (for *TNKS*) and melanoma, colorectal and prostate (for *TNKS2*, Fig. 3a and c). In the case of colorectal, bladder, hepatocellular and ovarian carcinoma alterations in *TNKS* expression are caused by deep deletion while in esophagogastric cancer *TNKS* gene amplification is responsible for most of the alterations in *TNKS*. Point mutations were responsible of *TNKS2* alterations for most tumors with exception of prostate adenocarcinoma and B-cell neoplasms (Fig. 3a and c). The reasons for the differences in the mutation mechanisms affecting *TNKS* or *TNKS2* differentially remain to be studied. Regarding the gene sequences, missense mutations were the most frequently found, followed by truncating, fusion and in-frame and they were distributed all along the genes (Fig. 3b and d). Overall survival of patients presenting altered expression in *TNKS* or *TNKS2* differed depending on the studies available in the public platform for both types of cancer, thus no conclusion can be drawn. High levels of TNKS1 and/or TNKS2 expression have been observed mainly in colon [92, 93] and lung cancer [39, 94], but also in brain cancer [95], breast cancer [96], ovarian cancer [97] and liver cancer [98]. The first TNKS inhibitor XAV939 was discovered in 2009 by Huang and colleagues while they were looking into Wnt/β-catenin pathway inhibitors [99]. Alterations in Wnt/β-catenin signaling have been observed in different tumors but, there are no direct inhibitors against this pathway in clinical use. For this reason, several targets of the Wnt/β-catenin pathway have been used to develop effective inhibitors. Porcupine inhibitors and anti-Frizzled antibodies, which block the secretion of Wnt ligands and prevent the ligand from binding to the receptor respectively, have been studied in clinical trials. Tankyrase inhibitors have been also developed as a therapeutic alternative to treat cancer, although there are no current clinical trials studying them [100–103]. These inhibitors recognize the catalytic domain and can be categorized according to the subsite of the donor NAD^+^ site they target: nicotinamide subsite (NS) (XAV939, AZ1366, RK-582 and LZZ-02), adenosine subsite (AS) (IWR1, G007-LK, OD366, OM-1700 and K756) or dual binders (DB) if they recognize both nicotinamide and adenosine subsites (TNKS656) (Table 2). The nicotinamide subsite is highly conserved among the PARP family while the adenosine subsite is more specific of tankyrases. This fact allows those inhibitors that target adenosine subsite present high selectivity towards TNKS [102, 116]. In the next section we will update the information about the role of tankyrases in cancer as well as the recent approaches using TNKS inhibitors as single and combined antitumor therapies. According to the current literature, colon and lung cancer have been two of the main models used for testing TNKS1/2 inhibitors (maybe due to the high percentage of alterations in the Wnt/β-catenin signaling components). For this reason, we will tackle the information about the inhibitors from the point of view of the cancer model in which they have been tested, highlighting the achievements and the issues that arose after their use in each model.

**Table 2:** Tankyrase-targeted anti-tumor options

TNKS inhibitor	Target	Type of cancer	References
**XAV939**	NS	Ovarian Breast Neuroblastoma Hepatocellular	[97] [96, 104] [105] [98, 106]
**IWR1**	AS	Lung	[94]
**G007-LK**	AS	Colon Glioblastoma Hepatocellular Melanoma	[107] [108] [106] [109]
**OD366**	AS	Colon	[110]
**OM-1700**	AS	Colon	[111]
**K-756**	AS	Colon	[102]
**AZ1366**	NS	Lung	[112]
**TNKS656**	DB	Lung Hepatocellular	[113] [114]
**RK-582**	NS	Colon	[115]
**LZZ-02**	NS	Colon	[116]

#### Colon carcinoma

Colorectal cancer is the third most common cancer and the second leading cause of cancer death worldwide [117] . The Wnt/β-catenin signaling pathway is hyperactivated in approximately 90% of human colorectal tumors most frequently due to the mutually exclusive mutation of tumor suppressor genes *APC* or *AXIN* or of the *CTNNB1* oncogene. These genetic alterations cause the accumulation of active β-catenin protein within the cell nucleus and the subsequent activation of its target genes that promote cell proliferation and malignancy [93, 101, 103, 115, 118]. Nowadays, TNKS inhibitors have gained attention due to its implication through the regulation of the limiting factor of the β-catenin degradation complex Axin [100, 102]. To date, many selective TNKS inhibitors with different structures and binding modes have been developed and used to treat colorectal cancer (Table 2).

##### G007-LK

G007-LK binds specifically to the adenosine subsite of the TNKS catalytic domain [119, 120]. This potent and selective inhibitor is able to induce the stabilization of Axin levels, which allows the stabilization of degradasomes in colon SW480 cell line. Degradasomes are structures formed by the components of β-catenin destruction complex which represent an active site for β-catenin turnover. The restoration of β-catenin degradation in APC-mutated cells after the treatment with G007-LK was shown, providing a link between TNKS inhibition and degradasomes formation in colorectal cancer cells [107]. Many new compounds have been synthesized from the chemical optimization of G007-LK. Two of them, OD366 and OM-1700 were deeply characterized and demonstrated good results and high selectivity towards TNKS1/2. Firstly, OD36 was obtained and showed a significant tumor size reduction after oral administration in the COLO 320DM xenograft immunodeficient mice model. Neither body weight loss nor animal discomforts was registered during the experimental period, although some adverse chemical properties required a new optimization, leading to OM-1700 identification. This new compound exhibited favorable parameters in general and reduced viability, proliferation and canonical Wnt/β-catenin signaling through Axin1/2 stabilization and active β-catenin reduction, as well as, the downregulation of β-catenin target genes [110, 111].

##### K-756

This inhibitor was found to recognize the adenosine subsite of TNKS and stabilize and reduce Axin and active nuclear β-catenin levels, respectively. Besides, K-756 was reported to induce cell growth inhibition in colon COLO 320DM and SW403 lines, whose proliferation is dependent on the Wnt/β-catenin signaling due to a short truncating mutation in APC component. The efficacy of K-756 was shown in an oral-administered DLD-1/TCF-Luc colon cancer xenograft mice model, proving the antiproliferative activity of K-756 [102].

##### RK-582

RK-582 binds to the nicotinamide subsite of tankyrases with a 200-fold selectivity against other PARP members and presents a remarkable level of efficacy at low doses. This new inhibitor blockades the Wnt/β-catenin pathway by elevating Axin2 and reducing active nuclear β-catenin levels while exhibits a potent tumor growth inhibitory effect in the mouse COLO-320DM xenograft model with oral or intraperitoneal administration [101, 115].

##### LZZ-02

The binding mode of LZZ-02 is similar to XAV939, since both recognize the nicotinamide pocket of TNKS. However, LZZ-02 forms more additional interactions with the residues near the border of the nicotinamide subsite in TNKS, which could improve the binding position and also enhance the inhibitory activity. LZZ-02 blockades the proliferation of APC mutated SW480 and DLD1 cells through the inhibition of the Wnt/β-catenin signaling. In addition, this inhibitor showed a robust antitumor effect in a DLD1-derived colon tumor xenograft model, pointing out the promising therapeutic application [116].

Despite tankyrase inhibitors have shown multiple benefits both in vitro and in vivo models; there are still remaining issues that have to be elucidated. Firstly, the status of the APC mutation, which has been considered as a potential biomarker for sensitivity to TNKS inhibitors in colon cancer [121]. Most colorectal tumors contain truncated APC mutations. Although all the mutations trigger the same phenotype (Wnt/β-catenin signaling hyperactivation), the mechanism underlying every mutation may cause different biochemical ways to respond to the same therapy [92]. According to the information obtained from K-756 and RK-287107, these inhibitors were able to induce cell growth inhibition in COLO-320DM and SW403 cells, but not in RKO, HCC2998 and DLD-1 cells. COLO-320DM and SW403 cell lines harbor “short-type” truncated APC mutations, which consist of lacking all the β-catenin-binding 20-amino acid repeats (20-AARs) and activate β-catenin signaling. Nevertheless, the other cell lines contain “long-type” truncated APC mutations, which partially preserve 20-AARs and activate β-catenin signaling modestly [101, 102]. The reason that could explain the different effects of TNKS inhibitors between both types of mutations is that Long-type mutations have been reported to confer resistance to tankyrase inhibitors due to a dominant negative effect on Axin2-dependent β-catenin degradation [121]. However, a recent report proved that long-type truncated APC mutation cells are also sensitive to TNKS inhibitors [92, 93]. In fact, the recently discovered TNKS inhibitor LZZ-02 has shown a potent therapeutic effect in a xenograft model derived from the DLD-1 colon cells [116]. All this information points out the need to get representative genetic models to define the genetic context, understand the molecular mechanism of a signaling pathway as well as achieve an accurate and effective treatment. Other option could be the use of TNKS inhibitors as a combination therapy to enhance their antitumor effect in colorectal cancer models that are resistant to TNKS inhibition. Solberg and colleagues proposed the use of G007-LK at low dosage with the PI3K (BKM120) and the epidermal growth factor receptor (EGFR) (erlotinib) inhibitors to treat colon cancer. The information obtained suggested that the activity of G007-LK was potentiated by the inhibition of both PI3K and EGRF pathways in the TNKS inhibitor-sensitive COLO-320DM and the inhibitor-resistant HCT-15 cell lines in vitro and in vivo tumor xenografts models [122]. Another important issue related to TNKS inhibitors is the balance between efficacy and toxicity. G007-LK, one of the most characterized TNKS inhibitors, was found to exhibit a strong tumor growth inhibition in colon xenograft mouse models. However, the efficacy of G007-LK was limited by the intestinal toxicity that carried out to reduced crypt proliferation and epithelial degeneration in the small intestine of mice [120]. RK-582 displayed an optimal toxicity profile in a xenograft mouse model administered orally or intraperitoneally. Weight loss and general toxicity were not observed at moderate dosage, although modest levels of intestinal toxicity were appreciated at high dosage, pointing out the unavoidable intestinal toxicity of TNKS inhibitors at high doses suggested by Zhong and collaborators [13, 115]. On the other hand, LZZ-02 was shown to have a potent antitumor effect as well as an extraordinary in vivo toxicity profile without body weight loss, which indicates that the inhibitor was well tolerated by mice [116]. These new inhibitors could become an effective cancer treatment, although the molecular mechanism requires additional investigation together with a better characterization in clinic.

#### Lung cancer

Lung cancer is the most diagnosed cancer and one of the foremost causes of cancer-associated mortality worldwide [123]. Non-small cell lung cancer (NSCLC) is the most common type of lung cancer, which encompasses around 80% of these cases [112]. Unlike colon carcinoma, abnormal Wnt activity in lung cancer is not only associated with APC or β-catenin mutations, but also a deregulation in the expression of genes associated with the canonical and non-canonical Wnt pathway. It has been found different components such upstream Wnt signaling effectors, for instance Dishvelled 3 (Dvl-3), or downregulation of Wnt antagonists as Wnt-inhibitory factor 1 (WIF 1) [94, 124]. In addition, TNKS1 and TNKS2 levels are elevated in lung cancer, as well as β-catenin levels [94, 123]. Another relevant pathway associated with some types of cancer is the Hippo-YAP (yes-associated-protein) pathway. It has been reported that alterations due to the activation in the Hippo kinase cascade trigger the phosphorylation of YAP, which is sequestered in the cytoplasm. When this cascade is not active, YAP can enter the nucleus and activate a downstream transcriptional program by the binding to TEAD family transcription factors. If this pathway is highly activated, it gives rise to an uncontrolled cell proliferation [113]. The effect of several TNKS inhibitors has been assessed in human and mice lung models (Table 2).

##### IWR1

IWR-1 has the ability to inhibit TNKS binding the adenosine binding pocket with a specificity of 100-fold than XAV939 for TNKS enzymes over other PARPs [99, 125]. The use of this inhibitor in human and murine lung cancer cell lines have provided a reduction in cell growth, as well as a decrease in the Wnt pathway, stabilizing protein levels of Axin and TNKS1/2 [94].

##### AZ1366

AZ1366 occupies the nicotinamide binding site of the PARP catalytic domain and works stabilizing Axin1 and decreasing the mRNA levels of different β-catenin targets. The most common subtype of NSCLC is associated with activating mutations in the EGFR gene. AZ1366 has shown a synergistic effect suppressing NSCLC proliferation in combination with EGFR inhibitors. When analyzing Wnt-responsive tumors in mice, an improvement in tumor control and survival using this combined inhibition was found compared to the single use of EGFR inhibitors [112].

##### TNKS656

This inhibitor is based on the optimization of XAV939, although it is consider a dual binder (DB) inhibitor since it binds both nicotinamide and adenoside subsites [126]. The efficacy of TNKS656 was demonstrated through the downregulation of nuclear YAP expression. This effect over YAP signaling is due to the angiomotin family proteins, which are tankyrase substrates. TNKS656 also proved a synergic effect with EGFR inhibitors in PC9, HCC827 and HCC4006 NSCLC cell lines through the inhibition of YAP signaling [113, 126, 127].

#### Other tumors

The role of tankyrases in the development of colon and lung cancer and the use of TNKS inhibitors are better known. Nevertheless, there is a growing knowledge concerning the use of TNKS inhibitors in other types of cancer (Table 2). The TNKS inhibitor XAV939 has been tested in ovarian cancer [97], breast cancer [96, 104], neuroblastoma [105] and hepatocellular carcinoma [98, 106]. It has been observed in these tumors that the use of XAV939 attenuates cell proliferation, viability by induction of apoptosis, migration, invasion and metastasis, as well as a decrease in aerobic glycolysis rate, which is one of the hallmarks of cancer. Besides, the use of XAV939 with the Polo-Like Kinase 1 (PLK1) inhibitor shown a synergistic effect concerning invasion, migration and proliferation, demonstrating the importance of PLK1 for TNKS1 stability [104]. The benefits of G007-LK have been reported in glioblastoma, proving a reduction in cell proliferation and sphere formation [108]. A decrease in cell growth and proliferation was also shown in hepatocellular carcinoma after the treatment of G007-LK combined with MEK and Akt inhibitors [106]. Moreover, the significance of the Wnt/β-catenin pathway and YAP signalling in the immune evasion has led to a combinatorial therapeutic strategy with the immune checkpoint inhibitor anti-PD-1 and G007-LK in melanoma, demonstrating the capacity to sensitize the tumor to the immune checkpoint therapy [109]. Recently, studies with the TNKS656 inhibitor in hepatocarcinoma cell lines gave evidences of suppression in cell proliferation, along with reduction in the levels of epithelial-mesenchymal transition (EMT) markers, invasion and metastasis [114].

#### New TNKS inhibitors targeting the ANK domain

All the current TNKS inhibitors are targeting the catalytic PARP domain. Tankyrase catalytic inhibition triggers the accumulation of many substrates and also TNKS1/2 [23]. Recently, it has been described a new catalysis-independent role of tankyrases as scaffolding proteins and the accumulation of tankyrases and their binding partners could promote the scaffolding mechanisms of both tankyrases [14, 31]. For this reason it has been explored different ways to develop new TNKS inhibitors against the ANK domain, which is responsible for the scaffolding function through the recognition and binding to the substrates of tankyrase. The strategy based on the binding mode of the fourth ARC of TNKS2 with the TBM of 3BP2 developed macrocyclized peptides that mimic the natural binding partners. Two of these peptides were able to disrupt the interaction with the substrates in vitro, and further showed the ability to enter cells and suppress the Wnt/β-catenin signaling through the disruption of TNKS-Axin interaction [128]. Another method based on a virtual screening was performed in order to find a molecule that could interrupt the interaction between tankyrase and the ubiquitin-specific protease 25 (USP25), which previously was shown to deubiquitinate and stabilize TNKS1/2 [129]. C44 was found to act as a protein-protein interaction (PPi) inhibitor that promoted the degradation of TNKS1/2, contributing to the inhibition of Wnt/β-catenin signaling via Axin stabilization and thus β-catenin degradation. C44 effectively decreased cell proliferation in the prostate cancer cell line PC-3, as well as it reduced cancer tumor growth in xenograft mice models [130]. Nowadays, two other different methods have been developed to find new peptidomimetics binding the ANK domain of tankyrase. One of them consists in a fragment-based screening using a combination of differential scanning fluorimetry (DSF) and nuclear magnetic resonance (NMR) spectroscopy [131]. The second one is an in vitro assay based on a fluorescence resonance energy transfer (FRET), which represents a reliable and cost-effective method to test compounds inhibiting the scaffolding function of tankyrase. Two molecules binding the ARC4 of TNKS2 with significant affinity were obtained using this method, indicating the potential therapeutic relevance of this new approach to find effective TNKS inhibitors [132].

## Conclusions

In view of the current scientific literature, there is still a long way to go to study in a more precise and systematic manner the implications of TNKS1/2 in the development of cancer. The role of tankyrases in the optimization of the Wnt/β-catenin pathway, key in the development of colorectal cancer and with implications in numerous types of cancer, is clear. Therefore, it is necessary to consolidate clinical studies and global data analysis to improve our current view of the role of TNKS1/2 in the origin of the alterations that lead to tumor development and the use of new therapeutic agents based on inhibition by tankyrases. In addition, it will be necessary in the future to deepen the knowledge of the TNKS interactoma in normal and tumor cells to define and identify new TNKS substrates. Future investigations will be also needed to elucidate the new functions of tankyrases that will allow to desing pharmacological inhibitors disrupting the TBM-ARC interaction independtly of the poly (ADP-ribosylation) activity.

## Data Availability

Not applicable.

## Abbreviations

3BP2: SH3 domain binding protein 2
AAR: Amino acid repeat
Akt: Protein kinase B
AMPK: AMP-activated protein kinase
ANK: Ankyrin
APC: Adenomatous polyposis coli
ARC: Ankyrin repeat cluster
ARTD: ADP ribosyl transferases diphtheria toxin-like
AS: Adenosine subsite
ATG9: Autophagy-related protein 9A
Axin1/2: Axis inhibition 1/2
Bcl-2: B-cell lymphoma 2
BRCA1/2: Breast cancer 1/2
CK1α: Casein-kinase 1 alpha
CTNNB1: Catenin Beta 1
DDR: DNA Damage Response
DSF: Differential scanning fluorimetry
DB: Dual binder
Dvl-3: Dishvelled
EGFR: Epidermal growth factor receptor
EMT: Epithelial-mesenchymal transition
FDA: Food and Drug Administration
FRET: Fluorescence resonance energy transfer
GMD: GDP-Mannose-4,6-Dehydratase
GSK3β: Glycogen synthase kinase 3β
HPS: His, Pro and Ser
IF4A1: Eukaryotic translation initiation factor 4A1
LKB1: Liver kinase B1
Mcl-1: Myeloid cell leukemia-1
MEK: Mitogen-activated protein kinase kinase
MERIT40: Mediator of Rap80 Interactions and Targeting 40 kD
MO25: Mouse protein 25
NAD: Nicotinamide adenine dinucleotide
NELFE: Negative elongation factor complex member e
NMR: Nuclear magnetic resonance
NS: Nicotinamide subsite
NSCLC: Non-small cell lung cancer
PAR: Poly (ADP-ribose)
PARdU: PAR-dependent ubiquitination
PARP: Poly (ADP-ribose) polymerase
PD-1: Programmed cell death-1
PEX14: Peroxin-14
PEX5: Peroxin-5
PI3K: Phosphatidylinositol 3-kinase
PLK1: Polo-Like Kinase 1
Pol-β: Polimerase-beta
PPi: Protein-protein interaction
PTEN: Phosphatidylinositol (3,4,5)-trisphosphate phosphatase
PTM: Posttranslational modification
RING: Really interesting new gene
RNF146: RING finger protein 146
SA1: Stromal antigen 1
SAM: Sterile alpha module
SCF: Skp, Cullin, F-box containing complex
SSB: Single Strand Break
SSBR: Single Strand Break Repair
STRAD: STE20 Related Adaptor Alpha
SUMO: Small Ubiquitin-like Modifier
TBM: Tankyrase binding motif
TCF: T cell factor
TEAD: Transcriptional enhanced associate domain
TIN2: TRF1-interactin nuclear protein 2
TNKS: Tankyrase
TRF1: Telomeric repeat-binding factor 1
USP25: Ubiquitin-specific protease 25
WIF 1: Wnt-inhibitory factor 1
WWE: Trp, Trp, Glu
XRCC1: X-ray repair crosscomplementing protein 1
YAP: Yes-associated-protein
β-TRCP: Beta-transducin repeats-containing proteins
